# Role of *KCNMA1 *gene in breast cancer invasion and metastasis to brain

**DOI:** 10.1186/1471-2407-9-258

**Published:** 2009-07-29

**Authors:** Divya Khaitan, Umesh T Sankpal, Babette Weksler, Edward A Meister, Ignacio A Romero, Pierre-Olivier Couraud, Nagendra S Ningaraj

**Affiliations:** 1 Department of Laboratory Oncology Research, Curtis and Elizabeth Anderson Cancer Institute, Hoskins Center for Biomedical Research, Savannah, Georgia 31404, USA; 2 Weill Medical College of Cornell University, Division of Hematology-Oncology 1300 York Avenue, New York, NY 10065, USA; 3 Clinical Research and Medical Education Department, Savannah, Georgia, USA; 4 Department of Sciences, The Open University, Walton Hall, Milton Keynes, UK; 5 Department of Cell Biology, Institut Cochin, 22 rue Mechain, 75015 Paris, France

## Abstract

**Background:**

The prognosis for patients with breast tumor metastases to brain is extremely poor. Identification of prognostic molecular markers of the metastatic process is critical for designing therapeutic modalities for reducing the occurrence of metastasis. Although ubiquitously present in most human organs, large-conductance calcium- and voltage-activated potassium channel (BK_Ca_) channels are significantly upregulated in breast cancer cells. In this study we investigated the role of *KCNMA1 *gene that encodes for the pore-forming α-subunit of BK_Ca _channels in breast cancer metastasis and invasion.

**Methods:**

We performed Global exon array to study the expression of *KCNMA1 *in metastatic breast cancer to brain, compared its expression in primary breast cancer and breast cancers metastatic to other organs, and validated the findings by RT-PCR. Immunohistochemistry was performed to study the expression and localization of BK_Ca _channel protein in primary and metastatic breast cancer tissues and breast cancer cell lines. We performed matrigel invasion, transendothelial migration and membrane potential assays in established lines of normal breast cells (MCF-10A), non-metastatic breast cancer (MCF-7), non-brain metastatic breast cancer cells (MDA-MB-231), and brain-specific metastatic breast cancer cells (MDA-MB-361) to study whether BK_Ca _channel inhibition attenuates breast tumor invasion and metastasis using *KCNMA1 *knockdown with siRNA and biochemical inhibition with Iberiotoxin (IBTX).

**Results:**

The Global exon array and RT-PCR showed higher *KCNMA1 *expression in metastatic breast cancer in brain compared to metastatic breast cancers in other organs. Our results clearly show that metastatic breast cancer cells exhibit increased BK_Ca _channel activity, leading to greater invasiveness and transendothelial migration, both of which could be attenuated by blocking *KCNMA1*.

**Conclusion:**

Determining the relative abundance of BK_Ca _channel expression in breast cancer metastatic to brain and the mechanism of its action in brain metastasis will provide a unique opportunity to identify and differentiate between low grade breast tumors that are at high risk for metastasis from those at low risk for metastasis. This distinction would in turn allow for the appropriate and efficient application of effective treatments while sparing patients with low risk for metastasis from the toxic side effects of chemotherapy.

## Background

A significant number of patients in the U.S with metastatic brain tumors face a dismal prognosis and high mortality. Increasing numbers of breast cancer patients are being diagnosed with brain metastases, possibly as a result of the emergence of targeted and aggressive systemic cancer therapy. In overall frequency, breast cancers and lung cancers are by far the most common cancers that metastasize to brain [1,2]. Brain metastasis generally arises in women diagnosed with aggressive breast cancer or in men with advanced lung cancer. The actual incidence of brain metastases is not precisely known, however studies suggest that 6–16% of patients with metastatic breast cancer develop brain metastases during their lifetime. Furthermore, autopsy studies have reported brain metastases in 18–30% of patients dying ofbreast cancer [3]. The majority of women who develop brain metastases have presented with debilitating neurological symptoms, and have undergone aggressive treatment for stage IV disease [4-6]. Although brain metastasis is the leading cause of breast cancer death, its pathogenesis is poorly understood and the predictors of breast metastasis to brain are yet to be characterized.

Accumulating evidence suggests that human epidermal growth factor receptor 2 (HER2) overexpression and consequent trastuzumab (Herceptin)-based therapy might be associated with a higher rate of brain metastases [7]. Retrospective studies in the U.S. in women with HER2 over-expressing breast cancer receiving trastuzumab-based treatment have indicated that approximately one-third of patients had developed brain metastases [7-9]. Palmeri et al. suggested that HER2 overexpression increases metastatic outgrowth of breast cancer cells in the brain [10], and that HER2 overexpression might be a predictor of asymptomatic, occult brain cancer. Therefore, it is extremely important to study the genetic changes in breast cancer cells that lead to brain metastasis and to develop specific targeted molecular agents. Many genetic aberrations are reported in human breast cancers, including altered splice variants and loss of genomic imprinting. Specifically, altered BK_Ca _channels, which respond to changes in intracellular calcium and membrane potential, are described in a wide variety of tumor cell types. For example, Huang [11] reported that p21^ras ^plays a pivotal role in controlling oncogenic transformation [12], and with its immediate downstream target, Raf kinase, is required for the induction of BK_Ca _channels. Although ubiquitously present in most human organs, the pore forming α-subunit of the BK_Ca _channel is encoded by the *KCNMA1 *gene, and is significantly unregulated and altered in cancer cells. In human glioma cells, up-regulation and constitutive activation of the *KCNMA1 *gene and its alternate splice variants are correlated with increased malignancy [13]. Up-regulation of BK_Ca _channels was shown to be a novel mechanism for the malignant phenotype of human tumor cells [14]. However, in osteosarcoma, *KCNMA1 *was shown to have antitumor property, suggesting that *KCNMA1 *may have diverse roles in different tumor types [15].

In the present study, our results from global exon array analysis showed higher expression of *KCNMA1 *in metastatic breast cancers located in brain than in metastatic breast cancers in other organs. This observation prompted us to investigate the potential for invasiveness and brain metastasis of *KCNMA1 *expression in normal breast cells (MCF-10A), non-metastatic breast cancer (MCF-7), non-brain metastatic breast cancer cells (MDA-MB-231), and brain specific metastatic breast cancer cells (MDA-MB-361). Our results clearly show that metastatic breast cancer cells exhibit increased BK_Ca _channel activity, leading to greater invasion and transendothelial migration. We showed that the invasion and transendothelial migration of breast cancer cells can be attenuated by blocking BK_Ca _channel activity. Therefore, determining the relative abundance of *KCNMA1 *in breast cancer metastases to brain and understanding its role in brain metastasis may allow us to target *KCNMA1 *for its prognostic and possibly therapeutic potential.

## Methods

### Cell lines and patient tissue samples

Established cell lines representing normal breast (MCF-10A), non-metastatic breast cancer (MCF-7, ER positive), non-brain metastatic breast cancer (MDA-MB-231, ER negative), and brain-specific metastatic breast cancer (MDA-MB-361, ER positive) were obtained from the American Type Culture Collection (Manassas, VA) and maintained as per ATCC guidelines. The established human brain microvasculature endothelial cell line (HCMEC/D3) of normal brain endothelial phenotype, kindly provided by Dr. Weksler (Weill Medical College, Cornell University, NY), was used to study transendothelial migration of the breast cancer cell lines [16]. Normal breast and breast tumor tissues were obtained from Memorial Health University Medical Center (MHUMC) and Comprehensive Human Tissue Network, University of Alabama according to the protocol approved by MHUMC Institutional Review Board.

#### Exon Array

Total RNA from exponentially growing breast cancer cell lines was extracted and purified using TRIzol reagent (Invitrogen) and RNeasy columns (Qiagen). Single-stranded cDNA was generated from the amplified cRNA with the WT cDNA Synthesis Kit (Affymetrix), fragmented and labeled with the WT Terminal Labeling Kit (Affymetrix). Samples were hybridized with GeneChip Human Exon 1.0 ST Arrays (Affymetrix) and scanned at the Memorial Health University Medical Center Genomics Core Facility. Intensity values were deposited with the GEO database http://www.ncbi.nlm.nih.gov/geo under the accession number GSE17019. Data was subjected to background correction and normalized using Expression Console (Affimetrix). Gene level expression values (probeset intensity analysis) were derived using RMA algorithm using core annotation level for transcripts. Exon array data was further analyzed using Partek Genomic Suite 6.4 software (Partek Inc., St. Louis, MO). Gene-level data was then filtered to include only those probesets that are in the 'core' meta-probe list, which represents RefSeq genes and full-length GenBank mRNAs. Analysis of Variance (ANOVA) and multi test correction for P-values in Partek Genomic Suite were used to identify differentially expressed genes, using tissue type (primary versus metastatic tissue) as the candidate variable in the ANOVA model to obtain metastatic specific genes.

### RNA extraction and PCR

Total RNA was extracted from cells which were 75% confluent and from the frozen tissues disrupted using a dounce homogenizer and extracted with TRIzol. The concentration of RNA was determined using a NanoDrop Spectrophotometer (NanoDrop Technologies, USA). cDNA for RT-PCR was generated by the SuperScript™ First-Strand Synthesis System according to manufacturer's instructions (Invitrogen). PCR was carried out in a total volume of 20 μl, containing 0.2 mM dNTPs, 1 mM MgCl_2 _and 1 Unit of AmpliTaq Gold DNA Polymerase (Applied Biosystem). Thirty two amplification cycles were performed (Applied Biosystem Thermal Controller), using a denaturing temperature of 95°C for 20 sec, an annealing temperature of 58°C for 30 seconds, and primer extension at 72°C for 20 sec. Each amplification experiment also included two negative PCR controls, a no-RNA control from reverse transcription procedures and a no-cDNA water control. Following amplification, 20 μl of the samples were separated via electrophoresis on a 2% agarose gel. The primers used for amplification of BK_Ca _channel were – (5'-TGCAAAGGAG GTTATAAAGTTACG-3' and 5'-ATTTCACAAAAGTTTTCACAAGGAC-3').

### BK_Ca _channel expression and localization in cell lines

Immunostaining of BK_Ca _channel protein was performed on paraformaldehyde fixed cells, which were permeabilized with 0.05% Triton-X 100 in Phosphate buffer saline (PBS) for 8 min on ice. After washes with PBS, cells were incubated with primary antibody (Santa Cruz Biotechnology, Inc., Santa Cruz, CA) diluted 1:400 in PBS containing 1% horse serum, 2% bovine serum albumin (BSA), and 0.05% Triton-X 100 for 30–45 minutes at 4°C. Cells were then washed with PBS containing 1% horse serum and 2% BSA, and incubated with fluorescein isothiocyanate (FITC)-labeled goat anti-rabbit IgG1 secondary antibody (Sigma) diluted 1:500 in PBS containing 1% horse serum, 2% BSA, and 0.05% Triton-X 100 for 60 min at 4°C. BK_Ca _channel green (FITC) fluorescence was recorded for a minimum of 5,000 events using a Guava flow cytometer. Images for localization of BK_Ca _channel protein were acquired using a Nikon Eclipse TE 2000U inverted microscope fitted with a fluorescence filter for FITC, Photometrics coolsnap HQ2 charge couple device camera, and Metamorph software.

### BK_Ca _channel expression and localization in tumor tissues

To test immunohistochemical expression of BK_Ca _channel α subunit protein, either fresh-frozen or paraffin-embedded tissues (breast metastasis to brain and one primary breast cancer tissue) were used. Fresh-frozen tumor specimens stored at -80°C were embedded in OCT (Tissue-Tek, Miles Laboratories, Elkhart, IN). Cryostat sections were cut at 10–12 μm thickness, mounted on lysine-coated glass slides, and fixed for 10 min at 4°C in 4% formaldehyde. This step was followed by a 5 minute rinse with PBS at pH 7.3. Slides were then blocked in blocking buffer (1% horse serum, 2% BSA, and 0.05% Triton-X 100) for 1 hour at 4°C to decrease nonspecific IgG binding. The sections were next incubated in a humid chamber at 4°C overnight with the primary antibodies, anti-BK_Ca _channel (rabbit) (Santa Cruz Biotechnology, Inc., Santa Cruz, CA) diluted 1:1000 in blocking buffer, or with PBS as a negative control. After 2 washes with PBS, the slides were stained with anti rabbit FITC conjugated secondary antibody.

To study BK_Ca _channel immunoreactivity in paraffin-embedded tumor specimens, sections were deparaffinized with three washes in xylene for 5 minutes each followed by rehydration with decreasing concentrations of ethanol. Following this, sections were heated in a water bath (30 minutes at 80°C) in citrate buffer for antigen retrieval. Then, they were washed in phosphate-buffered saline for 10 minutes and incubated with blocking buffer for 1 hour before the slides were incubated overnight at 4°C with the primary antibody rabbit anti-BK_Ca _channel diluted as above in blocking buffer or with PBS as a negative control. After 2 washes with PBS, the localization of BK_Ca _channels in the tissue sections was studied after staining with anti rabbit FITC conjugated secondary antibody.

Hematoxylin and eosin staining: Cryosections of breast tumors were stained with hematoxylin and eosin and examined by light microscopy.

### siRNA studies

The functional activity and biological role of *KCNMA1 *in breast cancer cells was studied by transiently knocking down the *KCNMA1 *gene using an Ambion Silencer siRNA transfection II kit. Transfection complexes were prepared in Opti-MEM serum-free medium (Invitrogen) by mixing 0.3 μL of siPORT NeoFX Transfection Reagent (Ambion) and 30 nM of siRNA (silencer pre designed siRNA AM16708A from Ambion). Breast cancer cells (8000 cells/well) were plated in 96-well plates simultaneously with addition of transfection complexes for membrane potential assays while 6-well plates (45,000 cells/well) were used for other studies. Cells were incubated for 72 h, and subsequently used for various assays.

### Membrane Potential Assay

The functional activity of BK_Ca _channels in breast cancer cells was measured using the FLIPR Membrane Potential Assay Kit on the FLEXstation (Molecular Devices, Sunnyvale, CA) as described previously [17]. This kit provided a fast, simple and consistent mix-and read procedure and has the potential to examine the functional characteristics of ion channels using changes in fluorescence. The BK_Ca _channel is one such ion channel, whose activation results in increase of membrane potential due to the opening of a K^+^-permeant, cation-selective channel leading to a decrease in fluorescence. Briefly, the cells were seeded in sterile, clear flat bottom, 96-well plates (Corning Inc., MA) at a density of 4 × 10^4 ^cells/well and cultured to achieve a monolayer within 48 hr. The monolayer of cells was incubated with the membrane potential assay reagents for 30 min before loading the test compounds. The anionic potentiometric dye that traverses between cells and extracellular solution in a membrane potential-dependent manner serves as an indicator of voltage changes across the cell membrane. Dose response studies were performed with 0 to 100 μM 1-(5-chloro-2-hydroxyphenyl)-5-trifluoromethyl-1,3-dihydro-2-benzimidazol-2-one (NS-004) (Neurosearch NA, Denmark) with or without Iberiotoxin (IBTX; 10 nM) (Sigma, St. Louis, MO). The FLEXstation was set up using the following parameters: excitation: 530 nm, emission: 565 nm and emission cut off: 550 nm wavelenghs. Observations and recordings were made for 300 seconds after adding NS-004 and IBTX.

### Matrigel Invasion

BD BioCoat™ growth factor reduced insert plates (Matrigel™ Invasion Chamber 12 well plates) were prepared by rehydrating the BD Matrigel™ matrix coating in the inserts with 0.5 ml of culture medium for two hours at 37°C. The rehydration solution was carefully removed from the inserts, 0.75 ml DMEM containing chemo-attractant (1%FBS) was added to the lower wells of the plate, and 0.5 ml of cell suspension (2.5 × 10^4 ^cells, in serum-free medium containing 0.1%BSA) was added to each insert well. For invasion inhibition assays IBTX (10 nM) was added to the cell culture medium in both upper and lower chambers along with cells and chemo-attractant solution. Cells transiently transfected with siRNA for the *KCNMA1 *gene was also plated to confirm our results obtained with IBTX. Untreated cells were included as migration controls, were used without rehydration. Invasion assay plates were incubated for 20–22 hours at 37°C. Following incubation, the non-invading cells were removed by scrubbing the upper surface of the insert. The cells on the lower surface of the insert were stained with crystal violet and each transwell membrane mounted on a microscopic slide for visualization and analysis. The number of tumor cells that had migrated from the upper to the lower side of the filter was counted by an individual blinded for the study using a phase-contrast microscope in the central area of the filter. Data are expressed as the percent invasion through the membrane relative to the migration through the control membrane.

% invasion = Mean number of treated cells invading through the matrigel insert membrane/Mean number of untreated cells migrating through membrane

### Transendothelial Migration

The invasion of cancer cells through a monolayer of brain endothelial cells is considered to represent the trans-cellular migration activity of the cancer cells. Basic insights into metastasis to brain have been obtained through experiments using human umbilical vein endothelial cells (HUVECs). However, endothelial cells from different vascular beds are uniquely adapted to meet the demands of the underlying tissues [18], and data from HUVECs are not directly applicable to breast tumor cell interaction with endothelial cells at the blood brain barrier (BBB). In this regard, differences between HBMVECs (human brain microvscular endothelial cells) and HUVECs have been reported [18]. We therefore used HCMEC/D3 cells, which are derived from human brain microvasculature to study transendothelial migration of breast tumor cells to brain. HCMEC/D3 cells were seeded at a density of 2 × 10^5 ^cells/dish on 35-mm plastic culture dishes coated with collagen and allowed to grow for 4 days to form a confluent monolayer. After the monolayer of HCMEC/D3 has been rinsed once with fresh culture medium, NS-004, IBTX and siRNA- treated breast cancer cells were seeded on top of the HCMEC/D3 at a density of 5 × 10^4 ^cells/dish. Following co-incubation for 16 hourat 37°C, non-adherent cells were removed by aspiration and the remaining cell layer was fixed in 10% formalin in PBS. The number of cancer cells that had penetrated the monolayer as well as cancer cell colonies (collectively called invasion foci) was counted under a phase-contrast microscope in 16 different visual fields. Data were expressed as percentage migration of treated breast cancer cells compared with migration of untreated controls, presumably metastatic to brain since the cancer cells were co-cultured with human brain microvascular endothelial cells.

## Results

### Exon array and PCR

**Figure 1:**
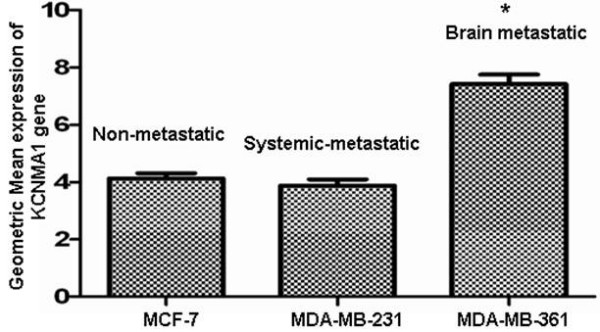
**Expression of *KCNMA1 *gene using Affymetrix Genechip Exon Array**. mRNA expression of *KCNMA1 *was significantly enhanced in the MDA-MB-361 (brain metastatic) cancer cell line as compared to the MCF-7 (non metastatic) and MDA-MB-231 (systemic metastatic) cell lines. MCF-7 (P < 0.0002) and MDA-MB-361(*P < 0.0001), (n = 3 per cell line).

**Figure 2:**
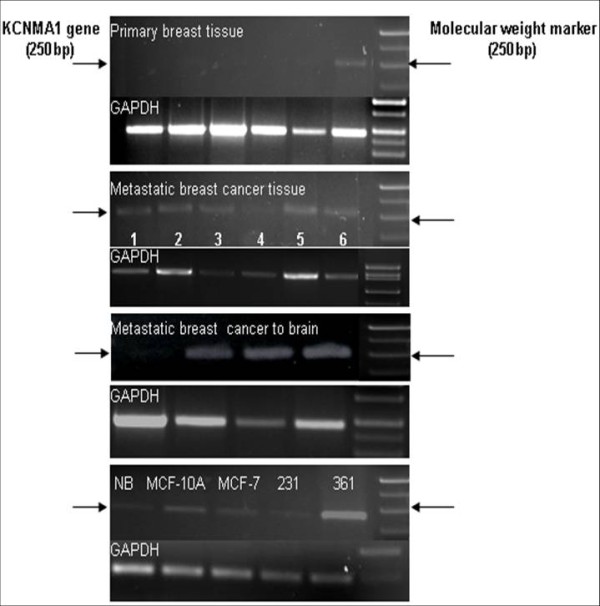
**Analysis of *KCNMA1 *gene expression by RT-PCR**. Semiquantitative RT-PCR analyses of human primary breast cancer (n = 6), metastatic breast cancer {n = 6, sample 1 (metastasized to ovary), 2 and 3 (metastasized to liver), 4–6 (metastasized to lymph node)}, metastasized to brain (n = 4) and breast cancer cell lines reveals differential expression of *KCNMA1 *gene expression (α-subunit, 250 bp). GAPDH was used as the loading control. NB: Normal breast tissue, MCF-10A (normal breast cell line), MCF-7 (non mets), 231: MDA-MB-231 (Mets) and 361: MDA-MB-361 (Brain Mets). Note: Only one of six primary breast cancer tissues showed detectable amount of *KCNMA1 *RNA (see arrow).

The data obtained by the GeneChip Exon array (Affymetrix) demonstrated that the *KCNMA1 *gene was over-expressed in breast cancer cell lines derived from human breast cancer metastases to brain (MDA-MB-361) as compared to metastatic breast cancer cells derived from systemic organs (MDA-MB-231) or primary breast cancer (MCF-7) (Figure 1). To study if differential expression of *KCNMA1 *occurs in *vivo *in different tissues, a series of specimens of fresh frozen and paraffin-embedded primary breast cancer, breast tumor metastases to different organs of the body and brain were analyzed by semi-quantitative RT-PCR. The PCR results were consistent with the microarray findings (Figure 2). Expression of the *KCNMA1 *gene was higher in breast tumor specimens from brain metastases compared to specimens from primary breast cancer or metastases to other organs. Similar results were also obtained using breast tumor cell lines of known metastatic predilection. These results imply that overexpression of *KCNMA1 *gene may likely be associated with breast cancer cells that metastasize to brain.

### Localization and expression of BK_Ca _channel protein

**Figure 3:**
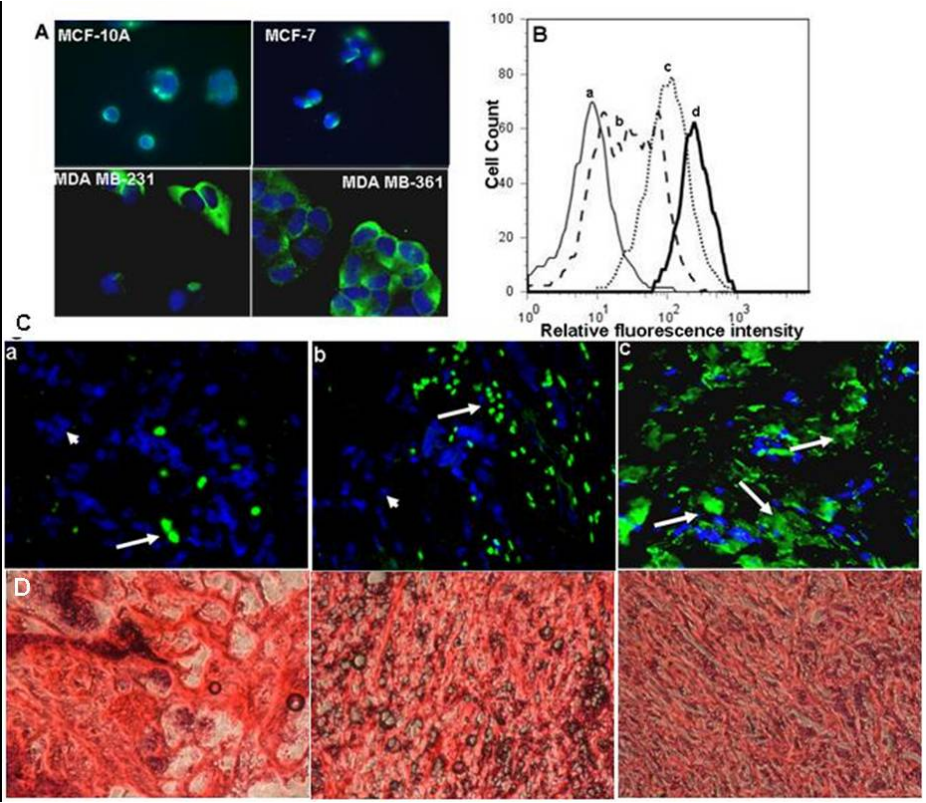
**Localization and expression of BK_Ca _channel protein**. A: Immunofluorescence of breast cancer cell lines stained for BK_Ca _channel (green) and nuclei with DAPI (blue). Expression of BK_Ca _channel was strong in MDA-MB-231 and MDA-MB-361, weak in MCF-7 and indiscernible in MCF-10A. Stains were repeated twice. B: Flow cytometric detection of BK_Ca _channel protein expression in various breast cell lines. Representative histograms from (a) MCF-10A, (b) MCF-7, (c) MDA-MB-231, and (d) MDA-MB-361 showing expression of BK_Ca _channel protein. C: Immunofluorescence of anti-BK_Ca _channels antibody on human breast cancer tissue. Antibody labeled in green (FITC), nuclei counterstained in blue (DAPI). (a) Very few cells show BK_Ca _channels staining in the primary breast cancer tissue (n = 6, See large arrow). Note: Absence of green staining in most of the cells also indicates specificity of the antibody (See small arrows). (b) Systemic metastatic breast cancer (lymph node) shows an increased number of cells stained with BK_Ca _channel antibody (n = 6; See large arrows). (c) All breast cancer tissue metastatic to the brain are strongly positive for the BK_Ca _channels (n = 4). Magnification = 400×. D: Representative H&E staining of breast tumor tissue sections.

Immunostaining was performed to localize BK_Ca _channel protein in breast cancer cells. Expression of the BK_Ca _channel was primarily confined to the plasma membrane (Figure 3A). Staining with affinity-purified anti-BK_Ca _channel antibody also revealed higher expression of BK_Ca _channel protein in MDA-MB-361 cells compared to MCF-10A, MCF-7 or MDA-MB-231 cells (Figure 3A). Control experiments with cells negative for BK_Ca _channels demonstrated lower protein signal intensity, indicating the specificity of the antibody (data not shown). In other supporting experiments, the mean fluorescence intensity (MFI) of BK_Ca _channel protein was 187 in MDA-MB-361 cells compared to 13 in MCF-10A, 46 in MCF-7, and 79 in MDA-MB-231 cells (Figure 3B). These results demonstrate the presence of different levels of BK_Ca _channel protein expression in different breast cancer cell lines evaluated.

Expression of BK_Ca _channel protein is not only restricted to cell lines but also present in human breast cancer tissues. Immunofluorescence performed on human breast cancer tissue revealed very few cells expressing BK_Ca _channel on primary breast cancer tissue (Figure 3C:a), while a moderate number of cells expressed BK_Ca _channel protein in breast cancer metastatic to different organs (Figure 3C:b). In agreement with the previous reports almost all the breast cancer cells metastatic to brain robustly expressed BK_Ca _channel protein (Figure 3C:c).

### Membrane potential

**Figure 4:**
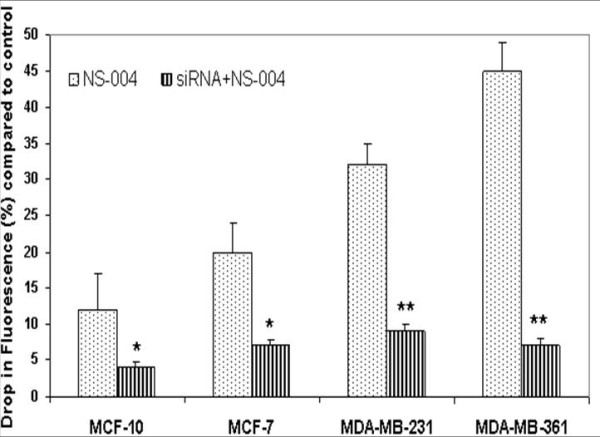
**Differential BK_Ca _channel activity measured by membrane potential in breast cancer cell lines**. The BK_Ca _channel activity was measured in the presence of its activator NS-004 with and without siRNA for *KCNMA1 *gene. Pretreatment of breast cancer cell lines with siRNA significantly (*p < 0.046; **p < 0.001) inhibited the BK_Ca _channel activity following addition of NS-004. *Note*: Decreased drop in fluorescence (indicator of BK_Ca _channel opening/activity) of siRNA + NS-004- treated cells compared to NS-004 treated cells demonstrates that BK_Ca _channel activity was inhibited by addition of siRNA to cells, as fluorescence quenching is an indirect measure of K^+ ^ion-mediated hyperpolarization (hyperpolarization is caused by efflux of K^+ ^through K^+ ^channels), shown in the Y-axis. Bars represent the mean and standard error of the mean obtained from three different experiments in triplicate (n = 9).

Immunolocalization and protein expression studies showed increased BK_Ca _channel expression in MDA-MB-361 cells as compared to MCF-7 or MDA-MB-231. We performed membrane potential studies to determine whether the overexpressed BK_Ca _channels show increased functional activity using the FLIPR Membrane Potential Assay kit on the FLEX station. Hyperpolarization is reflected as decrease in fluorescence at 565 nm. A specific BK_Ca _channel activator, NS-004, was added to the cells in a dose-dependent manner, and an optimum concentration of 50 μM was established (data not shown), for further studies. All the four breast cancer cell lines listed above showed hyperpolarization after the addition of NS-004 and the effect lasted for more than 300 seconds. The hyperpolarizing action of NS-004 was most marked in MDA-MB-361 cell lines (Figure 4) compared to other breast cancer cell lines. These results demonstrate that BK_Ca _channels in MDA-MB-361 and other breast cancer cell lines were functional. IBTX (10 nM), a BK_Ca _channel antagonist, blocked the membrane hyperpolarization in all the breast cancer cells tested. In addition, transient silencing of functional BK_Ca _channels by siRNA was performed. After testing different concentrations of siRNA (10–75 nM) 30 nM was determined to be optimum (>30 nM was toxic to cells, data not shown). Exposure of the breast cancer cells lines to 30 nM siRNA inhibited NS-004 induced activity of BK_Ca _channels by 65–70% in MCF-10A and MCF-7, and 75–85% reduction MDA-MB-231 and MDA-MB-361 cells (Figure 4, dark bars). These data indicate that BK_Ca _channel activity is functionally distinct in the four breast cancer cell lines studied, being highest in the line that metastasizes preferentially to brain, and can be silenced by a specific BK_Ca _channel inhibitor such as IBTX or by treatment with siRNA.

### BK_Ca _channel inhibition and effect on breast cancer cell invasiveness

**Figure 5:**
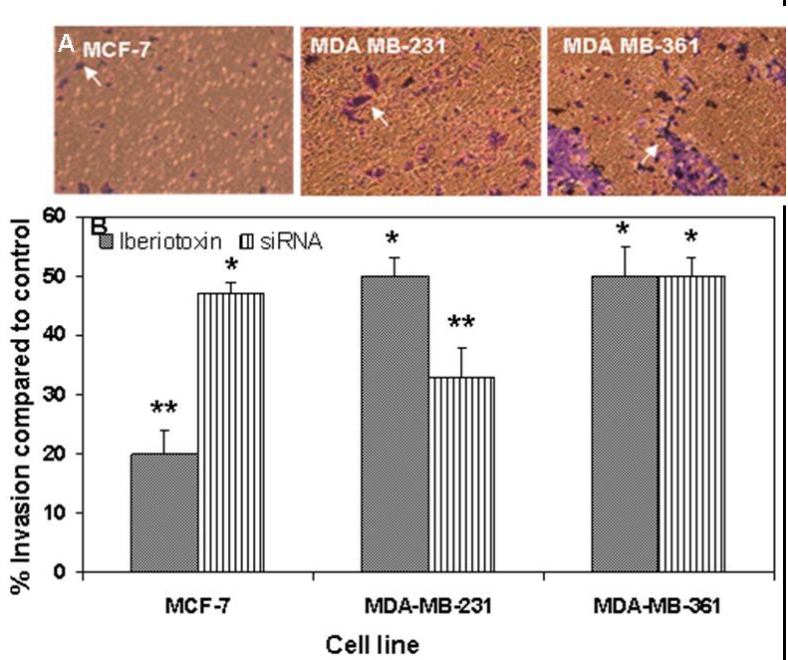
***KCNMA1 *expression promotes breast cancer cell invasion**. A: Representative images of breast cancer cells that have migrated through the Matrigel coated transwell and adhered to the lower surface of the filters. The migrated cells were stained with crystal violet (see arrows). B: Breast cancer cells pretreated with IBTX or siRNA to silence the *KCNMA1 *gene demonstrated significantly (*P < 0.03; **P < 0.001) reduced BK_Ca _channel mediated invasion compared to untreated breast cancer cell lines. Data are expressed as percentage of migration in the untreated controls (100%). Bars represent mean ± the standard error of the mean obtained from three different experiments (n = 3).

BK_Ca _channels are known to enhance the invasion of tumor cells. Therefore, we studied the effect of increased activity of BK_Ca _channels on the invasion of breast cancer cells into Matrigel-coated membranes in a Transwell chamber. No invasion was observed with MCF-10A cells, while MDA-MB-361 cells were more invasive than MCF-7 or MDA-MB-231 cells (Figure 5A). Interestingly the enhanced invasiveness of MDA-MB-361 cells was inhibited by only 50% by IBTX while invasion of MCF-7 was inhibited by 80% by IBTX (Figure 5B) suggesting that BK_Ca _channels may play a greater role in the invasion of MCF-7 cells. Furthermore, treating cells with siRNA that targeted the *KCNMA1 *gene inhibited the invasion of breast cancer cell lines to a similar extent as did treating them with IBTX (Figure 5B). These results clearly suggest that BK_Ca _channels may play a role in invasion of breast cancer cells because inhibition of BK_Ca _channels significantly attenuated the invasion process.

### Transendothelial migration (TEM) of breast cancer cells

**Figure 6:**
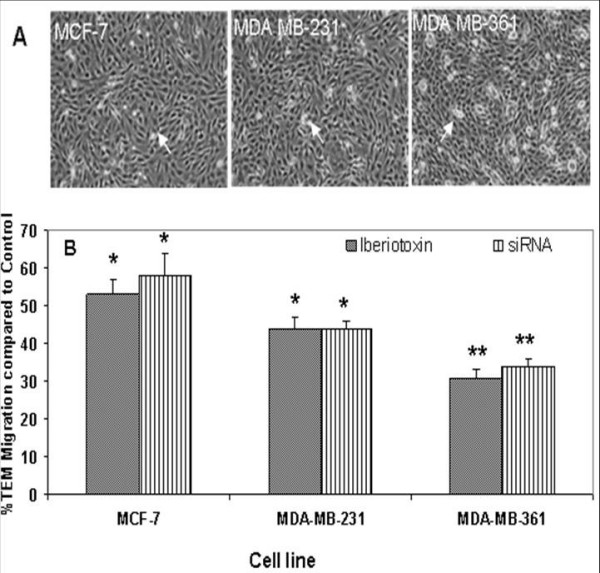
**Effects of IBTX and siRNA on transendothelial migration of breast cancer cells**. A: Representative images of breast cancer cells that adhered to monolayer of the human brain microvasculature endothelial cell line HCMEC/D3. The adherent breast cancer cells appear white in color against the background of HCMEC/D3. (See arrows). B: Breast cancer cells pretreated with IBTX or siRNA to silence the *KCNMA1 *gene demonstrated significantly (*P < 0.05; **P < 0.001) reduced BK_Ca _channel mediated-TEM of breast cancer cells across HCMEC/D3 monolayer compared to untreated control. Data is presented as percentage migration of treated breast cancer cells compared with migration of untreated controls (100%); Bars represent the mean ± standard error of the mean obtained from three different experiments (n = 3).

The effect of IBTX and siRNA on the capacity of breast cancer cells to invade a human brain microvascular endothelial cell monolayer (HCMEC/D3) *in vitro *was measured by a TEM assay as described by Kusama et al [19]. Non-brain metastatic breast cancer cells (MDA-MB-231) showed 65–70% less TEM than did brain metastatic breast cancer cells (MDA-MB-361) (Figure 6A), while normal breast epithelial cells (MCF-10A) did not show detectable TEM. IBTX inhibited the TEM of MCF-7 cells by 48% and migration of MDA-MB-361 cells by 69% (Figure 6B). The inhibition of TEM was similar when siRNA was used to suppress expression of the *KCNMA1 *gene (Figure 6B). These results clearly demonstrate that either chemical or biological inhibition of BK_Ca _channels might significantly decrease breast cancer metastasis to brain.

## Discussion

Genetic damage or alteration of primary tumor characteristics potentiates cancer metastasis to distant organs including brain. Tumor metastasis is a clonally selective process that occurs as part of tumor progression, a concept that has been supported by experimental [10] and clinical research. Generally, breast cancer metastasis is the hallmark of an aggressive tumor, resistant to treatment. Molecular factors that may contribute to organ-specific metastasis of breast cancer to lung and bone have been identified [20,21]. However, it is not clear how and why certain patients with primary breast cancers develop brain metastases, while in other patients tumors remain confined to the primary sites. Recent investigations in patients with metastatic lung and breast cancers reported alarming rates of brain metastases in both instances. For example, among metastatic breast cancer patients with HER2 positive tumors, the incidence of brain metastases varied from 26% to 48% (8–12). The brain and its supporting structures represent a unique environment for metastatic growth. Molecular factors contributing to the survival of tumor cells in metastatic lesions may be organ specific and could further influence the tumor cells with respect to their gene and protein expression, growth dynamics, and non-responsiveness to treatment. Unraveling the mechanism that drives the metastatic machinery in these cancer cells may offer insights into how to limit or potentially prevent this lethal process.

We showed that the biochemical opening of BTB with BK_Ca _channel modulators increases the BTB permeability and enhance delivery of hydrophilic therapeutic drugs or small- to large-sized molecules, anti-tumor compounds, therapeutic proteins and viral vectors *in vivo *to brain tumors selectively with little or no drug delivery to normal brain [17,22-24].

Increasingly, targeted molecular therapy is gaining popularity, especially the use of humanized monoclonal antibodies. Although targeting oncogenes expressed on tumor cells is one viable cancer treatment strategy, oncologists often need to target additional molecules related to tumor stroma that promote tumor metastasis and growth. We used gene expression profile study to identify increased *KCNMA1 *expression in a breast cancer cell line metastatic to brain (MDA-MB-361) (Figure 1) as compared to expression in a primary, breast cancer cell line (MCF-7) or breast cancer cell line metastatic to other organs (MDA-MB-231) of the body but not to brain. Others have shown an up-regulation of BK_Ca _channels as a novel mechanism for producing the malignant phenotype of human tumor cells [14]. Our results clearly demonstrate that the *KCNMA1 *is upregulated in the MDA-MB-361 cell line (Figure 1). RT-PCR data (Figure 2) from tumor tissues and from breast cancer cell lines further supported these findings, suggesting a close association between level of BK_Ca _channel expression and metastasis to brain. In agreement with the PCR results on breast cancer cell lines, immunocytochemistry results showed strong expression of BK_Ca _channels in MDA-MB-361 cells (Figure 3A) as compared to MCF-10A cells (Figure 3A). The presence of the BK_Ca _channel protein was also confirmed using flow cytometry (Figure 3B). The immuno staining of the sections from human breast cancer tissue indicated that BK_Ca _channel protein expression was pervasive in metastatic brain tumors (Figure 3C:c) compared to primary cancers and those metastatic to other organs (Figure 3C:a&b). In addition, we showed that BK_Ca _channel is pharmacologically functional by knocking down the *KCNMA1 *gene with siRNA, which has led to a decrease in activity (drop in fluorescence) in all the cell lines studied (Figure 4). The BK_Ca _channel activation was 3-fold higher in breast cancer cells metastatic to brain (MDA-MB-361) than in normal breast cells (MCF-10A) (Figure 4). These results confirm higher expression of BK_Ca _channels on brain specific metastatic breast cells (MDA-MB-361) than in normal breast cells and non-brain metastatic breast cancer cells (MDA-MB-231). These findings corroborate the immunostaining data (Figure 3A) and are consistent with our previous studies showing overexpression of BK_Ca _channels in gliomas and in endothelial cells surrounding malignant brain tumors [17]. Other investigators have also demonstrated over expression of BK_Ca _channels to correlate with increased malignancy of human gliomas [25]. Other type of K^+ ^channel (KCNK9) was also shown to have oncogenic properties in breast cancer [26]. Taken together our data suggest that there may be an association between BK_Ca _channel overexpression and breast cancer metastasis to brain.

The most recent electrophysiological information about BK_Ca _channels came from studies on vertebrate cells with the patch clamp [27]. The cDNAs of BK_Ca _channels have been cloned, and its 7-transmembrane topology has been identified. The N-terminus of BK_Ca _channels is in the extracellular space because an additional transmembrane segment called S0 precedes the canonical S1 through S6 transmembrane segments. With fixed level of intracellular Ca^2+ ^[Ca^2+^]_i_, BK_Ca _channels behave like delayed-rectifier K^+ ^channels, activating with depolarization where the gating rates and the resulting probability of being open are also sensitive functions of [Ca^2+^]_i_.

Various therapeutic suggestions have been made by investigators based on genome-wide expression analysis of breast cancers, such as adjusting the intensity of current chemotherapy based on cancer subtypes as defined by gene expression signatures [28]. Unfortunately, because a large number of genes with many diverse functions are identified as prognostic signatures, the lists of genes may yield excellent prognostic markers without revealing much of the underlying biological mechanisms thereby providing little guidance for a course of action. In contrast our study showed that inhibition of BK_Ca _channels using either a siRNA or specific chemical inhibitor significantly reduces *in vitro *the invasive capability of the breast cancer cell lines (Figure 5). This finding further requires validation in biological tumor models. Surprisingly the decrease in the invasive capability of MCF-7 following the addition of BK_Ca _channel inhibitor IBTX was greater than that observed in MDA-MB-361 cells. One possible explanation could be that MDA-MB-361 cells are HER2 positive, which may induce the breast cancer cells to metastasize to brain. Furthermore, HER2 expression may skew the BK_Ca _channel induced invasiveness of MDA-MB-361 cells, while MCF-7 (HER2 negative) cells may depend predominantly on BK_Ca _channels for invasion.

The unique properties of the brain may explain the propensity of certain breast cancer cells to metastasize to the brain. Specifically the brain vascular endothelial cells forming the BBB are significantly different from the systemic endothelial cells [29]. A key event in brain metastasis is TEM of breast cancer cells. We examined whether inhibition of BK_Ca _channels expressed in breast cancer cells metastatic to brain, prevents TEM of MCF-7, MDA-MB-231, and MDA-MB-361 cells across the monolayer of HCMEC/D3 cells. As anticipated, targeting *KCNMA1 *with siRNA or BK_Ca _channels with IBTX significantly decreased the TEM of MCF-7, MDA-MB-231, and MDA-MB-361 cells across the monolayer of HCMEC/D3 (Figure 6). Most importantly a normal breast cell line (MCF-10A) did not exhibit TEM even when the BK_Ca _channels were activated with NS-004 (data not shown). Our data suggest that increased TEM, at least in part, requires increased BK_Ca _channel expression and/or activity. Thus, blocking BK_Ca _channel function potentially may delay or prevent breast cancer metastasis to the brain. In summary, we showed that BK_Ca _channels are overexpressed in breast cancer cells metastatic to brain. The high expression level correlated with BK_Ca _channel activity, invasion and TEM of the breast cancer cells metastatic to brain.

## Conclusion

Tumor metastasis is a complex and highly regulated process involving multiple tumor-host interactions. Accurately determining breast cancer's risk of metastasis to other organs including brain will improve the capacity of clinicians to decide on optimal patient management. Identifying organ-specific *KCNMA1 *gene involved in metastatic process, and developing targeted therapies against *KCNMA1 *will hopefully lead to better treatments for this deadly metastatic disease. Furthermore, advances in the identification of contributing factors including the role of *KCNMA1 *in the metastatic processes, as well as understanding its function and regulation may result in the development of targeted agents to suppress BK_Ca _channel over expression in breast cancers.

## Abbreviations

BBB: Blood-brain barrier; BK_Ca_: maxi calcium-activated voltage sensitive potassium; *KCNMA1*: BK_Ca _channel α-subunit gene; CCD: Charge couple device; PBS: Phosphate-buffered-saline; PCR: Polymerase chain reaction; MgCl_2_: Magnesium chloride; FITC: Fluorescein isothiocyanate; FLIPR: Fluorescent Imaging Plate Reader; NS-004: 1-(5-chloro-2-hydroxyphenyl)-5-trifluoromethyl-1,3-dihydro-2-benzimidazol-2-one; IBTX: Iberiotoxin; DMEM: Dulbecco's Modified Eagle's Medium; MFI: Mean fluorescence intensity; CNS: Central nervous system; TEM: Transendothelial migration; MHUMC: Memorial Health University Medical Center.

## Competing interests statement

The authors declare that they have no competing interests.

## Authors' contributions

DK contributed 50% in all aspects of this work. UTS helped in the exon array and PCR studies. BW, IAR and POC provided the human brain microvasculature endothelial cells and helped to draft the manuscript. EAM performed statistical and microarray data analyses. NSN participated in conception, design, and coordination of the study and helped drafting the manuscript. All authors read and approved the final manuscript.

## Pre-publication history

The pre-publication history for this paper can be accessed here:

http://www.biomedcentral.com/1471-2407/9/258/prepub
